# Caring for the elderly: A person-centered segmentation approach for exploring the association between health care needs, mental health care use, and costs in Germany

**DOI:** 10.1371/journal.pone.0226510

**Published:** 2019-12-19

**Authors:** Beate Wild, Dirk Heider, Dieter Schellberg, Friederike Böhlen, Ben Schöttker, Dana Clarissa Muhlack, Hans-Helmut König, Joris Slaets

**Affiliations:** 1 Department of General Internal Medicine and Psychosomatics, Medical University Hospital, Heidelberg, Germany; 2 Department of Health Economics and Health Services Research, University Medical Center Hamburg-Eppendorf, Germany; 3 Division of Clinical Epidemiology and Aging Research, German Cancer Research Center, Heidelberg, Germany; 4 Departments of Internal Medicine and Geriatrics, University Hospital, Groningen, Netherlands; Chiba Daigaku, JAPAN

## Abstract

**Background:**

Person-centered care demands the evaluation of needs and preferences of the patients. In this study, we conducted a segmentation analysis of a large sample of older people based on their bio-psycho-social-needs and functioning. The aim of this study was to clarify differences in health care use and costs of the elderly in Germany.

**Methods:**

Data was derived from the 8-year follow-up of the ESTHER study—a German epidemiological study of the elderly population. Trained medical doctors visited n = 3124 participants aged 57 to 84 years in their home. Bio-psycho-social health care needs were assessed using the INTERMED for the Elderly (IM-E) interview. Further information was measured using questionnaires or assessment scales (Barthel index, Patients Health Questionnaire (PHQ) etc.). The segmentation analysis applied a factor mixture model (FMM) that combined both a confirmatory factor analysis and a latent class analysis.

**Results:**

In total, n = 3017 persons were included in the study. Results of the latent class analysis indicated that a five-cluster-model best fit the data. The largest cluster (48%) can be described as healthy, one cluster (13.9%) shows minor physical complaints and higher social support, while the third cluster (24.3%) includes persons with only a few physical and psychological difficulties (“minor physical and psychological complaints”). One of the profiles (10.5%) showed high and complex bio-psycho-social health care needs (“complex needs”) while another profile (2.5%) can be labelled as “frail”. Mean values of all psychosomatic variables—including the variable health care costs—gradually increased over the five clusters. Use of mental health care was comparatively low in the more burdened clusters. In the profiles “minor physical and psychological complaints” and “complex needs”, only half of the persons suffering from a mental disorder were treated by a mental health professional; in the frail cluster, only a third of those with a depression or anxiety disorder received mental health care.

**Conclusions:**

The segmentation of the older people of this study sample led to five different clusters that vary profoundly regarding their bio-psycho-social needs. Results indicate that elderly persons with complex bio-psycho-social needs do not receive appropriate mental health care.

## Introduction

Over the past years the proportion of elderly people in many countries has increased dramatically. These demographic changes go hand in hand with an increase in the prevalence of chronic diseases; this presents a challenge for health care systems [1]. To date, the highly heterogeneous group of older people accounts for a disproportionate share of both services and costs in health care [2–4].

Because of the high heterogeneity of this group the approach to “older people” as a single group is not helpful if one wishes to understand and improve health care use. Diagnostic and disease-related categories are therefore frequently used to understand and explain the relationship between quality of health care and costs for specific subgroups [5, 6]. However, this approach frequently results in a fragmented and provider based understanding of health care. Disease-related approaches usually do not provide information on the needs of various patients or the intensity of required treatment. In order to provide an optimal balance between quality and costs health care, one requires a person-centered approach [7], in which health care providers try to incorporate the perspectives of patients [8–10]. As a result, person-centered care emphasizes the limitations of a disease-centered approach and asks for an evaluation of the needs, values, and preferences of patients [11]. The focus of this approach lies not on the cure of chronic illnesses, but rather on the improvement—and even optimization—of both the functioning and the quality of life.

A segmentation model, based on the specific health care needs of the elderly, could reduce heterogeneity by providing meaningful clusters from the point of view of the individuals concerned—not from the point of view of professionals with particular diagnostic categories. Because costs are directly related to delivered care we believe that the relationship between the clusters and the costs could be very informative.

To date, only a few previous studies exist that have attempted to segment elderly people into more homogeneous subgroups [2, 12–14]. Most of these studies identified cure-oriented clusters by basing their segmentation analysis on the measurement of chronic conditions [2, 12, 13]. However, the difficulties experienced by the elderly are determined not only by their diseases, but also by their psychosocial situation.

In 2014, for the first time, Eissens et al. [14] published a person-centered segmentation study of the elderly Dutch population based on their self-assessed bio-psycho-social health care needs. To date, such an analysis does not exist in Germany. Moreover, no study has yet compared the health care use of the elderly in two different European countries that is based on the same segments. Using the frame of the ESTHER study [15], we built up a data-base of elderly people that contains variables very similar to the variables used in the Dutch segmentation study. This presents us with the possibility to segment the ESTHER population according to the same (adapted) variables as used in the Dutch study. By using a segmentation model that is based on the same variables we can explore and compare the efficacy of the two health care systems on a population-based level in a further study.

The overall aim of this particular study was to clarify the differences and variation in both health care use and costs of the elderly in Germany. Specifically, we wanted to use a person-centered approach in order to describe, explain, and compare health care use and costs in relation to the subjective bio-psycho-social needs and functioning of elderly people. As the group of elderly people is very heterogeneous we first conducted a segmentation analysis according to thie bio-psycho-social needs and functioning in order to define more homogenous subgroups. Subjective needs and functioning profiles, together with a distribution of the elderly across the profiles, were determined. Differences between profiles regarding health care use and costs are described in the following.

## Methods

### Study population

The data was derived from the 8-year follow-up of the ESTHER study—a population-based cohort study of older adults conducted in the federal state of Saarland, Germany [16]. The study was approved by the ethics committees of the University of Heidelberg and of the medical board of the state of Saarland, Germany. Written informed consent was obtained from all participants. At the baseline of the ESTHER study (between July, 2000 and December, 2002) 9940 participants were recruited by their general practitioners in the course of a health check-up that is offered biennially to older adults in Germany. Between 2008 and 2010, 6063 elderly people participated in the 8-year follow-up. All participants of the 8-year follow-up were asked if they would take part in a longer home visit to be conducted for personal interviews and a geriatric assessment. Out of the 6063 ESTHER participants, n = 3124 persons agreed to be visited at home. The home visits served as a comprehensive assessment tool regarding functional status as well as the medical, pharmacological, socio-economic, and psychosocial aspects of their life. At the conclusion of the home visit, the INTERMED for the Elderly (IM-E) was conducted to assess the bio-psycho-social health care needs of the participants [17].

### Assessment instruments

#### Indicators for the segmentation analysis

The segmentation analysis was based on the functioning and bio-psycho-social needs according to Eissens et al. [14]; indicators for the physical, psychological, social, mobility, and cognitive domains were used. In the ESTHER study, the following instruments that provided indicators for these five domains were applied: the INTERMED for the Elderly (IM-E) interview, the Barthel Index, and the Lubben Scale (LSNS-6) for social network. Cognitive impairment was measured by using three basic questions.

The INTERMED for the Elderly (IM-E) interview is based on the original INTERMED interview. It is altered and adjusted specifically for application in elderly populations [17]. Previous work showed that the IM-E is a reliable integrative assessment instrument; it is well suited for epidemiological settings [17]. The IM-E classifies the information into the four domains of a patient’s biological, psychological, social, and health care related characteristics. In each domain the questions are related to a time axis that is divided into past, present, and future. The answers of the individual questions are scored by means of a four-level rating scale, ranging from 0 to 3; the range encompasses the spectrum of zero evidence for a symptom, disturbance, or health service need (0) to evidence of complex symptoms or health service needs (3). The respective scores of the four domains are added to give a total score ranging from 0 to 60; the total score reflects the amount of health care needs of the participant.

The short Lubben scale (LSNS-6) is a concise instrument that measures social isolation in older adults. It consists of six items that assess the level of perceived support received from family, friends, and neighbors [18]. The LSNS-6 was shown to have a high internal consistency and a good convergent validity. The scores of the items range between 0–5, added to give a total score.

The Barthel index is a tool for assessing self-care and mobility activities of daily living. The ten activities that are evaluated include feeding, wheelchair/bed transfer, personal care, toilet transfer, bathing, walking, stair climbing, and dressing as well as bladder and bowel control. The scores of the items are totalled with a maximum score of 100 [19]. The validity of the Barthel index in different contexts is reported in Lübke et al. [20].

Self-perceived cognitive impairment in the ESTHER study was assessed by the question “I’ve often been misplacing things lately “(0 = no; 1 = yes, sometimes; 2 = yes, often, always).

#### Additional variables to describe the clusters

The Generalized Anxiety Disorder Scale (GAD-7) was applied to assess symptom severity of the generalized anxiety disorder (GAD). The total score of the GAD-7 ranges from 0 to 21. A previous study of our working group showed that the German version of the GAD-7 proved to be a valid instrument for the screening of GAD in elderly people [21].

Depression was assessed using the 8-item Patient Health Questionnaire depression scale (PHQ-8) [22] that consists of eight of the nine DSM-IV diagnostic criteria of a major depressive disorder. The ninth criterion, assessing suicidal thoughts, is omitted—a common practice in population-based studies [23]. Scores of the PHQ-8 range from 0 to 24, with higher scores indicating a higher depression severity. The sensitivity (98%) and specificity (80%) of the PHQ depression module proved to be excellent when using the Structured Clinical Interview for DSM-IV as criterion standard [22].

Somatic symptom severity was measured using 13 items from the PHQ-15 questionnaire [24]. The PHQ-15 is comprised of 15 somatic symptoms (stomach pain, back pain, chest pain, etc.); each symptom was scored from 0 to 2. The PHQ-15 is a widely used measurement instrument with well-established validity and reliability [25, 26]. In this study, only 13 items were applied, problems regarding menstruation and sexual intercourse were omitted.

Health service utilization of the participants was assessed using a short version of a questionnaire that had been applied in several previous studies [16, 27, 28]. Data on health service utilization was collected retrospectively for three months. Frequency, type, and duration/quantity of health service utilization were recorded and included the following variables: inpatient care, treatment at day clinics and rehabilitation, outpatient treatment by physicians and other therapists, pharmaceuticals, medical supplies, and dental prostheses. Costs were calculated from the societal perspective by multiplying resource use with unit costs for the year 2009, with unit costs reflecting average prices of the German healthcare system [29]. Hospital care costs, for instance, were calculated by valuing days in hospital with unit costs for stationary hospital care (515.81 €), stationary psychiatric care (261.54 €) and stationary rehabilitation (106.88 €). For example, a fictive person spending 5 days in stationary hospital, 2 days in stationary psychiatric care, and 10 days in rehabilitation would have caused individual hospital care costs of 5 x 515.81€ + 2 x 261.54€ + 10 x 106.88€ = 4170.93 € during the three-months period of our study.

### Statistical analysis

The segmentation analysis applied a factor mixture model (FMM) that combined both a confirmatory factor analysis and a latent class analysis. The purpose of such a model is to test whether a sample is combined of several subpopulations (i.e. classes), each having its own unique characteristics [30]. The FMM is an extension of the common factor model [31].

In the first phase of the analysis confirmatory factor analyses (CFA) were conducted including five factors (one for each domain: physical, psychological, social, mobility, and cognitive), using diverse sets of variables. The aim of the CFAs was to determine the best set of variables representing the five different domains. The final item set consisted of 16 items: five items from the INTERMED interview regarding physical needs, four INTERMED items regarding psychological needs, two items of the Lubben scale for the social needs, four items of the Barthel index regarding mobility, and one item regarding cognitive impairment. The resulting item set is displayed in detail in Supporting information 1. For each factor (domain) a mean value was built so that each study participant had a total of five factor values.

In the next step, the common factor model was extended with a latent class analysis to model unobserved population heterogeneity. To identify the number of classes the model was fitted multiple times; the number of classes specified was varied each time [30]. The models were then compared on a variety of fit indices (Akaike information criterion (AIC), the likelihood-ratio goodness-of-fit value L^2^ etc.). According to these indices the best model was then chosen. After the decision was made on the number of classes, the probability of belonging to each of the classes was predicted for each participant using multinomial regression. Subsequently, each elderly person was assigned to the segment for which he/she had the highest probability. Finally, the resulting segments were described regarding (a) demographic variables, (b) bio-psycho-social variables, (c) health care use, and (d) costs. Most of the results were reported using descriptive statistics. Comparisons of percentages between different clusters were tested using chisquared-tests, while mean values between clusters were compared using ANOVAs or independent t-tests.

For the latent class analysis, Latent Gold version 5.1 was used. For all other statistical analyses, SAS version 9.4 was used.

## Results

In total, n = 3017 persons were included in the cluster analysis. Of these, n = 107 persons had to be excluded due to missing values in one of the indicator variables used.

**Fig 1:**
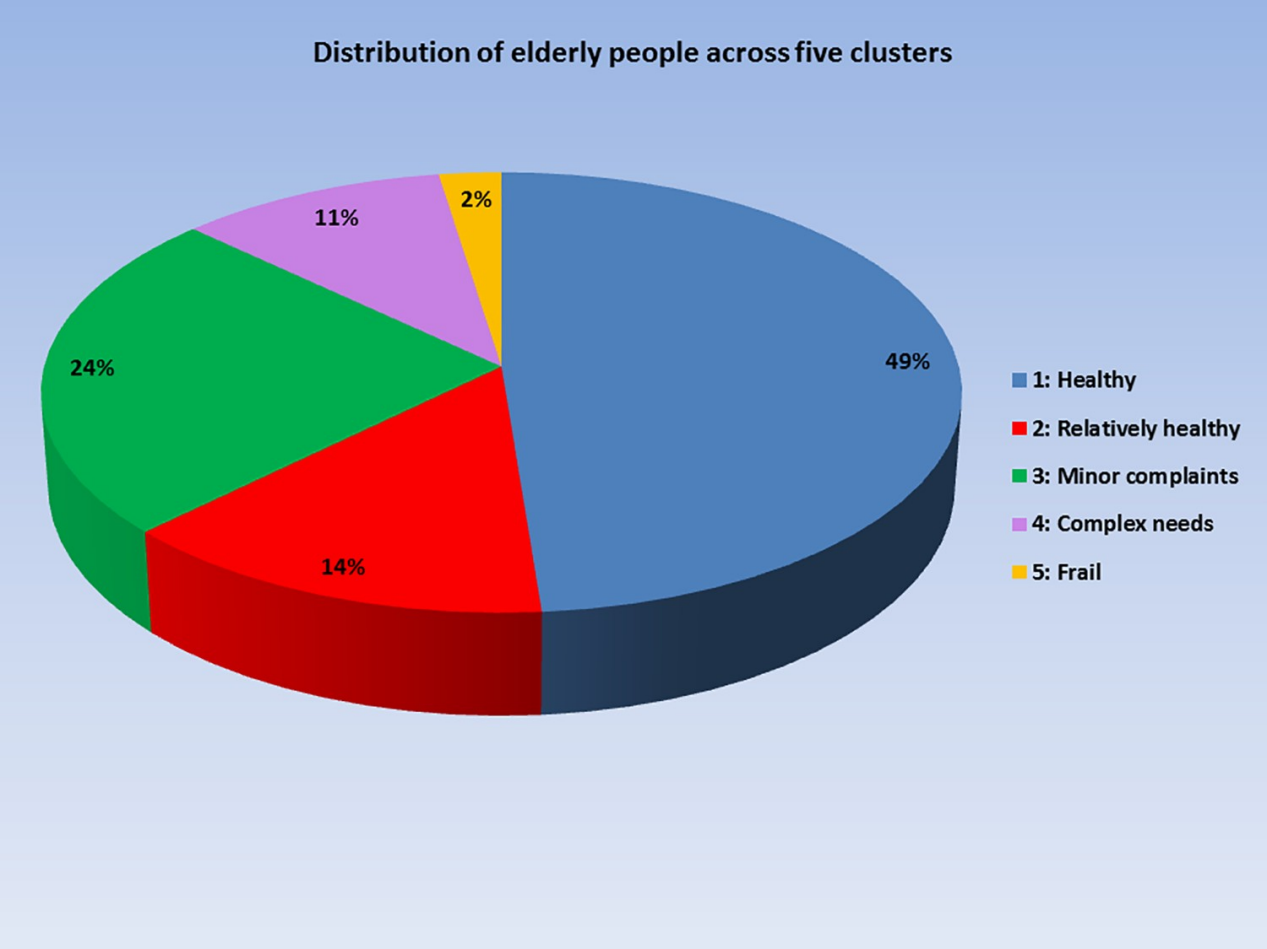
Distribution of elderly people across five clusters.

Supporting information 2 shows the various cluster solutions of the latent class analysis together with their fit indices. The L^2^ statistics (with a p-value >0.05) and the AIC criteria suggest that the 5-cluster solution would best fit the data (L^2^ = 3045.4; p = 0.086; AIC = -2834). A conditional bootstrap test to assess the significance in the difference in the L^2^ statistics associated with the 4- and 5-cluster-solutions showed that the 5-cluster-model provided a significant improvement over the 4-cluster-model (p = 0.018). Fig 1 displays the distribution of the elderly across the five clusters, while Table 1 shows the demographic characteristics of all five clusters.

**Table 1:** Demographic characteristics of the five clusters of elderly people.

Demographic variable	Healthy	Minor physical complaints	Minor physical and psychological complaints	Complex needs	Frail
	(n = 1471) (48.7%)	(n = 420) (13.9%)	(n = 734) (24.3%)	(n = 318) (10.5%)	(n = 74) (2.4%)
	n (%)	n (%)	n (%)	n (%)	n (%)
Gender					
Male	722 (49.1)	244 (58.1)	333 (45.4)	92 (28.9)	40 (54.1)
Female	749 (50.9)	176 (41.9)	401 (54.6)	226 (71.1)	34 (45.9)
Age (years)					
57–64	384 (26.1)	89 (21.2)	167 (22.8)	96 (30.2)	12 (16.2)
65–74	773 (52.6)	225 (53.6)	379 (51.6)	153 (48.1)	33 (44.6)
75–84	314 (21.4)	106 (25.2)	188 (25.6)	69 (21.7)	29 (39.1)
Median Age	69	70	71	69	72
Marital Status					
Single	44 (3.0)	13 (3.2)	23 (3.2)	18 (5.7)	2 (2.7)
Married	1042 (71.8)	299 (72.9)	520 (71.4)	228 (72.1)	51 (68.9)
Divorced	115 (7.9)	31 (7.6)	47 (6.5)	22 (7.0)	5 (6.8)
Widowed	251 (17.3)	67 (16.3)	138 (19.0)	48 (15.2)	16 (21.6)
Education (years)					
≤ 9	1087 (75.3)	287 (70.5)	509 (71.0)	232 (73.9)	60 (83.3)
10–11	215 (14.9)	63 (15.5)	126 (17.6)	47 (15.0)	8 (11.1)
≥ 12	141 (9.8)	57 (14.0)	82 (11.4)	35 (11.1)	4 (5.6)
	**Mean (SD)**	**Mean (SD)**	**Mean (SD)**	**Mean (SD)**	**Mean (SD)**
Factor 1 Psychological domain	0.20 (0.28)	0.21 (0.31)	0.54 (0.43)	1.44 (0.52)	0.95 (0.62)
Factor 2 Physical domain	0.78 (0.42)	1.26 (0.24)	1.72 (0.28)	1.69 (0.38)	1.96 (0.31)
Factor 3 Mobility	1.00 (0)	1.01 (0.07)	1.00 (0)	1.00 (0.03)	1.39 (0.46)
Factor 4 Social domain	2.55 (0.76)	4.17 (0.51)	3.19 (0.81)	2.35 (0.80)	4.51 (0.48)
Factor 5 Cognition	0.41 (0.51)	0.62 (0.56)	0.45 (0.52)	0.99 (0.55)	0.77 (0.59)

In the physical, psychological, mobility, and cognitive domains higher values indicate an increased number of complaints, greater needs, or severity of their condition. In the social domain, higher values indicate more support.

With the exception of the frail cluster the age distribution is quite similar across the clusters: In the frail cluster the percentage of persons aged 75–84 is significantly higher compared to the other clusters (ANOVA; F_4;3012_ = 6.98; p<0.0001)). The gender distribution is significantly different between the clusters (χ^2^_4_ = 989.1; p<0.0001) with the “complex needs” cluster having the highest percentage of women (71.1%). Apart from the social domain, the mean values on the five factors used for the segmentation analysis show that the largest cluster is the “best” or healthiest cluster in all areas—that is, people are healthy and independent (“healthy”). Compared to the healthy cluster, cluster 2 exhibits a somewhat stronger impairment in the physical domain, but more social support in everyday life—the seniors show minor physical complaints and high social support (“minor physical complaints”). One cluster (24.3%) could be characterized with “minor physical and psychological complaints”. Persons in a smaller cluster (10.5%) have major physical, psychological and cognitive complaints and low social support (“complex needs”). Seniors in the smallest cluster (2.4%) show the highest impairment in the physical and mobility areas but have more everyday life support (most likely in the form of care giving in contrast to the social support in the cluster with minor physical complaints); they show major physical and mobility complaints and high social support (“frail”).

Table 2 further illustrates the bio-psycho-social characteristics of all five clusters by showing the distribution of mean values in additional variables that were not used for the cluster identification.

**Table 2:** Bio-psycho-social characteristics of the five clusters of elderly people.

	Healthy	Minor physical complaints	Minor physical and psychological complaints	Complex needs	Frail
	(n = 1471) (48.7%)	(n = 420) (13.9%)	(n = 734) (24.3%)	(n = 318) (10.5%)	(n = 74) (2.4%)
	Mean (SD)	Mean (SD)	Mean (SD)	Mean (SD)	Mean (SD)
Health care needs (total INTERMED score)	6.9 (3.7)	10.8 (2.6)	15.6 (3.4)	19.9 (4.5)	21.3 (4.2)
Biological domain score (INTERMED)	3.5 (2.5)	6.3 (1.3)	8.6 (1.4)	8.4 (1.9)	9.8 (1.6)
Health care use (INTERMED domain score)	2.1 (1.4)	3.2 (1.5)	4.0 (1.6)	4.4 (1.8)	5.4 (1.6)
Frailty Index (according to Fried)	0.89 (0.84)	0.90 (0.91)	1.17 (1.07)	1.34 (1.10)	2.39 (1.41)
Physical quality of life (PCS)	44.4 (8.5)	41.9 (8.8)	36.7 (9.5)	36.1 (9.0)	28.1 (8.3)
Mental quality of life (MCS)	49.8 (7.9)	50.2 (7.0)	47.3 (9.7)	37.8 (10.8)	40.2 (10.7)
Somatic complaints (PHQ-13)	3.6 (3.0)	4.3 (3.3)	6.8 (4.0)	9.5 (4.2)	10.5 (4.9)
Depression severity (PHQ-9)	1.6 (2.1)	1.9 (2.4)	3.2 (3.1)	6.4 (4.3)	7.2 (4.7)
Generalized anxiety severity (GAD-7)	1.2 (1.8)	1.2 (1.8)	2.4 (2.8)	5.3 (4.5)	5.0 (4.7)

PCS = Physical Component Scale; MCS = Mental Component Scale; PHQ = Patient Health Questionnaire; GAD-7 = Genaralized Anxiety Disorder questionnaire

In general, the mean values of all additional variables gradually increased across the five clusters, with the highest complexity and burden occurring in the frail cluster. Similarly, quality of life decreases across the five clusters, with the highest physical (PCS) and mental quality of life (MCS) in the healthy cluster and the lowest in the frail cluster. However, the highest mean value in the generalized anxiety disorder (GAD) severity in the “complex needs” cluster together with the high mean INTERMED total score (19.9), reflect the first characterization of this cluster as having high bio-psycho-social difficulties and health care needs.

Table 3 shows the percentage of persons per cluster experiencing a depression or anxiety disorder and self-reported mental health care use.

**Table 3:** Distribution of mental disorders and mental health care use in the five different clusters.

	Healthy	Minor physical complaints	Minor physical and psychological complaints	Complex needs	Frail
	(n = 1471) (48.7%)	(n = 420) (13.9%)	(n = 734) (24.3%)	(n = 318) (10.5%)	(n = 74) (2.4%)
	N (%)	N (%)	N (%)	N (%)	N (%)
Depressive disorder (PHQ ≥ 10)	9 (0.61)	8 (1.9)	40 (5.5)	74 (23.3)	20 (27.0)
Generalized anxiety disorder (GAD >10)	7 (0.48)	2 (0.48)	18 (2.5)	40 (12.6)	10 (13.7)
Depressive disorder and/or GAD	16 (1.1)	10 (2.4)	50 (6.8)	89 (28.0)	23 (31.5)
Treatment by a psychologist or psychiatrist (in the past 3 months)	23 (1.6)	7 (1.7)	20 (2.7)	43 (13.5)	7 (9.5)
	**Mean (SD)**	**Mean (SD)**	**Mean (SD)**	**Mean (SD)**	**Mean (SD)**
Costs in € spent for outpatient mental health care (over past 3 months)	1.1 (13.7)	0.6 (7.2)	2.6 (33.8)	25.0 (131.1)	4.7 (20.2)

A χ2 –test was used to compare percentages of depressive/GAD disorders between clusters: “Complex needs” vs. “minor complaints”: χ^2^_1_ = 86.8, p<0.0001; “frail” vs. “minor complaints”: χ^2^_1_ = 48.2, p<0.0001.

In the complex and frail clusters the percentage of depression and/or anxiety disorders is markedly and significantly higher compared to the healthier clusters. Accordingly, the percentage of elderly persons who used mental health care is also higher in the complex and frail clusters than in the healthier clusters. However, in the cluster “minor physical and psychological complaints” as well as in the “complex needs” cluster, only half of the elderly with a mental disorder were treated by a mental health professional; in the frail cluster only one third of those with a depression or anxiety disorder had received mental health care.

Table 4 shows the inpatient and outpatient costs (per three months) for the five clusters.

**Table 4:** Inpatient and outpatient costs in the various clusters.

	Healthy	Minor physical complaints	Minor physical and psychological complaints	Complex needs	Frail
	(n = 1471)	(n = 420)	(n = 734)	(n = 318)	(n = 74)
	Mean € (SD)	Mean € (SD)	Mean € (SD)	Mean € (SD)	Mean € (SD)
	[min; max]	[min; max]	[min; max]	[min; max]	[min; max]
Inpatient costs	250.60 (1275.3)	475.23 (2226.5)	480.98 (1901.3)	457.68 (1843.1)	1253.98 (4799.3)
	[0; 18 054]	[0; 23 312]	[0, 19 085]	[0, 17 688]	[0; 30 994]
Outpatient costs	346.03 (527.8)	411.74 (560.0)	567.44 (690.8)	556.13 (585.6)	877.04 (845.7)
	[0; 10 456]	[0; 5 430]	[0; 8 379]	[0; 8 379]	[76;4 902]
Total cost per person	**596.63** (1432.7)	**886.97** (2382.0)	**1048.42** (2107.3)	**1013.81** (1962.1)	**2131.02** (4943.2)
	[0; 18 554]	[0; 23 571]	[0; 20 193]	[0; 16 970]	[75, 31 113]
Total cost per cluster (three months)	**877 642.73**	**372 527.4**	**769 540.28**	**322 391.58**	**157 695.48**

Mean € are given for a three-months period

In the frail cluster, mean total costs per person are four times higher than in the healthy cluster, and twice as high as in the cluster experiencing complex needs. However, as the frail cluster is quite small and the healthy cluster is large, the total costs spent in the frail cluster are about 5.5 times lower compared to the costs spent in the healthy cluster. Interestingly, in each cluster (even in the healthy cluster) there are persons with very high total costs (Please see the maximum of the costs in Table 4).

**Fig 2:**
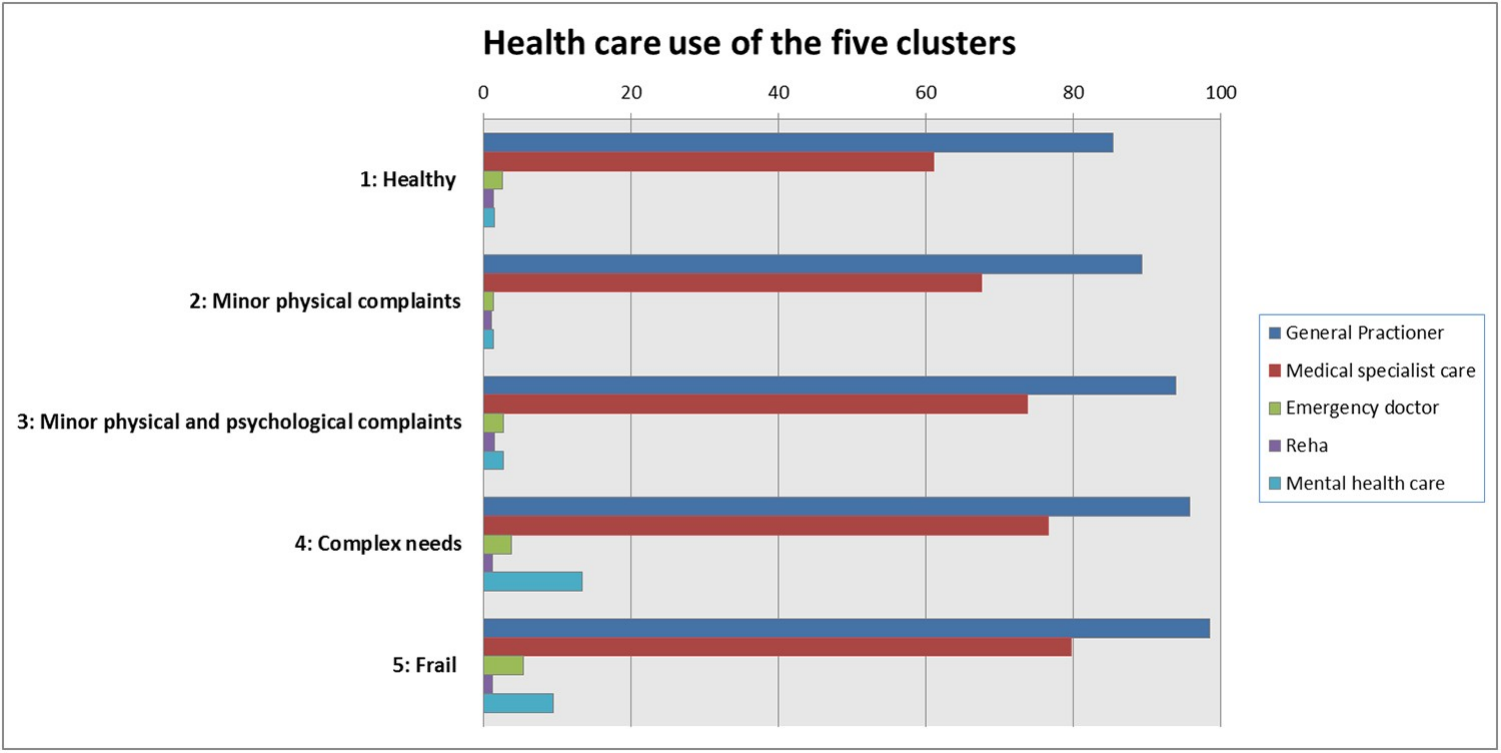
Health care use of the five clusters.

Fig 2 shows the health care use in various sectors of the five clusters.

## Discussion

In this segmentation analysis a person-centered approach was used to cluster a large group of older people based on their bio-psycho-social needs. Results indicate that older people can be meaningfully clustered into five relatively homogenous groups. This segmentation provides a structure for the heterogeneous population of elderly people, thereby making it easier to recognize patterns and relationships in health care use.

Compared to the clusters found in the Netherlands, the various clusters could be characterized very similarly, although not equally [14]. Similar to the Dutch study, a five-cluster-solution provided the best data fit. It is interesting to note that in both countries a large cluster was identified that could be characterized as healthy and independent. Further, in both of these countries a smaller cluster that included patients with complex health care needs and a very small cluster with frail older persons were identified. However, it should be pointed out that the percentages of persons per cluster differ widely in the corresponding clusters of the two study samples. This is explained by the fact that the study sample from the Netherlands included about 40% retirement and nursing home residents whereas this large segment of the older population was not included in the ESTHER study.

In our study, the largest cluster includes 48% of the ESTHER participants and can be described as healthy and independent (“healthy”). Persons in a smaller cluster (13.95) show, on average, higher somatic difficulties but are still considered healthy, differing from the other clusters because of high social support (“minor physical complaints”). Another cluster includes persons with only a few physical and psychological complaints (“minor physical complaints”); in comparison to the healthy cluster and the cluster with minor physical complaints, this cluster shows a decrease in mental health, although not a dramatic one. One profile appears to be the cluster with complex health care needs (10.5%) composed of persons who show major physical, psychological and cognitive complaints and low social support. The smallest cluster (2.4%) includes the frail elderly people who are extremely burdened in each domain. Mean values of all psycho-somatic variables either increase (e.g. depression severity, anxiety severity) or decrease across the clusters (e.g. health related quality of life). This can be seen as proof for the content validity of the segmentation analysis.

According to the PHQ depression module and the GAD-7 questionnaire the distribution of mental disorders also changed dramatically across the clusters. Compared to 1.1% in the healthy cluster and 6.8% in the cluster with minor physical and psychological complaints, approximately 30% of the persons in the complex and frail cluster had a depression and/or generalized anxiety disorder. Interestingly, in the healthy cluster more than 1.1% of persons had received mental health care in the past three months; in contrast, the complex and frail clusters appear to be clearly undertreated by mental health professionals. To date, many studies have shown that depression and anxiety disorders in older people often go undetected and undertreated [21, 32, 33]. However, the segmentation analysis enabled us to pinpoint the most disadvantaged groups in regard to mental health care, namely the complex and frail elderly people. This could partly be explained by the high somatic impairment and needs of the complex or frail elderly people. In these groups it appears that health care providers focus more on medical treatment and do not consider—or are not aware of—the psychosocial needs of the patients. One clinical implication of this observation could be that a better diagnosis of the psycho-social needs of complex and frail people is needed, together with integration into treatment approaches. One step towards this goal could be the use of more holistic assessments or narratives of the older people in order to acknowledge their personal life-history and to offer person-centered appropriate support. In addition, for caregivers, the possibility of offering older people who suffer from high psychosomatic burden psychotherapeutic treatment could be more present. It is well known that older people can greatly benefit from psychotherapeutic approaches [34]; however, this idea still appears to be abandoned in clinical practice.

Another interesting result of the segmentation analysis is the distribution of total costs spent in health care across the clusters. Based on the decreasing health outcomes over the various clusters, it is fair to assume that persons in the complex and frail clusters would cause the highest mean costs. However, there are also persons in the healthier clusters who show relatively high health care costs.

Regarding health care use in specific sectors, it should be noted that the use of rehabilitation treatment and emergency care is similarly distributed across the clusters. Rehabilitation treatment in the healthy cluster and the cluster with minor physical complaints could be seen as a preventive strategy; however, the use of emergency care appears relatively high in the healthier clusters compared to the same percentage in the more burdened clusters.

If the various clusters were to be classified in regard to their risk profile, one could reduce the number of clusters in accordance with other approaches. Several years ago Uittenbroek et al. [10, 35] conducted an RCT to evaluate the efficacy of person-centered, integrated care of older people. They used the same instruments as used in this study to assess the risk profiles of the participants. They classified the older people into three risk profiles “Robust”, “Complex care needs”, and “Frail” [36]. The “Robust” profile included adults without complex care needs and a low frailty level. Presumably, the first three clusters of our study would correspond to this “robust” profile while our complex cluster would correspond to the profile “Complex care needs” and the frail cluster to the risk profile “Frail”. It is obvious that in terms of risk factors and health care needs, the complex and frail clusters are more highly burdened compared to more robust clusters. Uittenbroek et al. [35] targeted the high risk groups in their intervention study, and therefore defined three risk profiles. However, our cluster analysis indicates that a five—cluster solution would best fit the data. Clusters 1–3 are clearly healthier compared to the complex and frail clusters, but still show various differences which should be considered when planning stepped care interventions or prevention strategies.

Our study has several limitations: firstly, we investigated health care use and costs by using personal reports of the participants and not by using insurance data; costs could therefore have been underestimated. However, we assume that an underestimation of costs would be equally applicable to all participants, in which case the relationship between costs and clusters would still be valid. A second limitation is that our study did not include elderly people living in retirement or nursing homes. In the ESTHER study the comprehensive home visits were conducted with those participants that were still residing at home. The study sample of the home visits of the third follow-up of the ESTHER study is thus not representative for the general population of this age. Nevertheless, the internal relationships between bio-psycho-social health care needs and costs are valid in our study sample.

## Conclusions

Our study provides evidence that the segmentation of elderly people, based on their bio-psycho-social needs, is a useful and important approach for investigating certain aspects of health care systems such as sufficiency of mental health care or need for a better coordination of care. Gaps between health care needs and care delivery (costs) can be identified and the distribution of health care resources in relation to personal needs can thereby be improved. By using this approach one could also measure the effects of interventions and changes in reimbursement systems. In the usual normative system it is very difficult to measure the effects of preventive interventions related to life style and psycho-social support [37, 38]. For example, from a patient-centered perspective, the transition to a better segment in a single year could be a relevant outcome measure for an intervention. It could thus be interesting for health insurances to use such a segmentation analysis for the planning and improvement of health care.

## Supporting information

S1 File Item set used for the final factor analysis. (DOCX)

S2 File Comparison of model fit statistics. (DOCX)
