# Supplementary material for: Caring for the elderly: A person-centered segmentation approach for exploring the association between health care needs, mental health care use, and costs in Germany
Source: PLoS One. 2019 Dec 19;14(12):e0226510. doi: 10.1371/journal.pone.0226510 (PMC6922348; doi:10.1371/journal.pone.0226510)
Supplement: S1 File — (DOCX) [file pone.0226510.s001.docx]

**S1: Item set used for the final factor analysis**

**Physical needs (INTERMED)**

1. Which of your physical illnesses have been ascertained over the last 5 years? As a result, did you feel negatively impacted for a longer period of time (more than 3 months)?

- Less than 3 months of physical dysfunction
- More than 3 months of physical dysfunction or several periods of less than 3 months
- A chronic disease
- Several chronic diseases

2. How difficult has it been over the past five years to diagnose an illness or a disease?

- No illness that required a more detailed diagnosis
- Diagnoses and etiology were easily clarified
- Diagnostic dilemma solved, but only with considerable diagnostic effort
- Diagnostic dilemma; not solved despite considerable diagnostic effort

3. Do you have any physical complaints or restrictions at this time? If so, how have these complaints affected you over the last week?

- No symptoms or mild symptoms that do not interfere with current functioning
- Mild but noticeable symptoms that interfere somewhat with current functioning
- Moderate to severe symptoms that clearly interfere with current functioning
- Severe symptoms that lead to an inability to perform functional activities

4. At this time, do you have physical complaints or restrictions that have still not been resolved or dealt with in a satisfactory manner?

- No diagnostic problems / clear diagnosis
- No clear diagnosis at this time, but a prompt clarification of the cause of the complaints can be expected
- Complex differential diagnosis; somatic origin of the complaints is to be expected
- Complex differential diagnosis; additional psychological aspects are to be expected

5. Prognosis of the interviewer for the next 6 month regarding “complications and life threat”, based on the information obtained.

- No limitations in activities of daily living
- Mild limitations in activities of daily living
- Chronic condition and/or substantial permanent limitations in activities of daily living
- Severe physical complications and functional deficits, serious risk of dying

**Psychological needs (INTERMED)**

6. How did you cope with physical restrictions or stressful, difficult situations over the course of your lifetime?

- No restrictions in coping; ability to manage stress adequately, no impairment of medical treatment
- Mild restrictions in coping, e.g. anxiety, volatility or denial without adverse consequences for the person
- Moderate restrictions in coping that cause severe emotional distress, such as depressive mood and denial that could lead to self harm (e.g. not taking medication; not attending regular check-ups)
- Severe limitations in coping that produce serious psychiatric symptoms (such as substance abuse, severe depression, or attempted suicide)

7. Over the course of your lifetime, have any psychiatric disorders been determined? If yes, did you feel negatively affected as a result?

- No psychiatric dysfunction
- Psychiatric dysfunction without clear effects on daily functioning
- Psychiatric dysfunction with clear effects on daily functioning
- Psychiatric admission(s) and/or permanent effects on daily functioning

8. From an emotional point of view, how have you been feeling over the course of the last week? Have you ever seen a psychiatrist or have there been periods when you were anxious, depressed, or confused?

- No psychiatric symptoms
- Mild psychiatric symptoms such as problems concentrating or feeling tense
- Psychiatric symptoms such as anxiety, depression or confusion
- Psychiatric symptoms with behavioural disturbances such as violence, obvious confusion, or total withdrawal from social interaction

9. Prognosis of the interviewer for the next 6 months regarding “mental health threat”, based on the information obtained

- No psychiatric disorder
- Mild psychiatric disorder such as adjustment disorder, anxiety, feeling depressive or cognitive disturbance
- Moderate psychiatric disorder requiring psychiatric or psychosomatic outpatient care
- Severe psychiatric disorder requiring psychiatric or psychosomatic admission

**3) Mobility Needs (Barthel Index)**

10. Variable “Toilet use” from the Barthel Index of Activities of Daily Living

- Dependent
- needs some help, but can do something alone
- independent (on and off, dressing, wiping)

11. Variable “Mobility” from the Barthel Index

- immobile or < 50 yards
- wheelchair independent, including corners, > 50 yards
- with help of one person (verbal or physical) > 50 yards
- independent (but may use any aid; for example, stick) > 50 walks yards

12. Variable “Dressing” from the Barthel Index

- dependent
- needs help but can do about half unaided
- independent (including buttons, zips, laces, etc.)

13. Variable “Feeding” from the Barthel Index

- No, I don’t think this is difficult
- needs help cutting, spreading butter, etc., or requires modified diet
- independent

**4) Social needs (Lubben Network Scale)**

14. How often does somebody support you in everyday life?

- seldom
- sometimes
- often
- daily
- several times daily

15. How often does somebody show you love and affection?

- seldom
- sometimes
- often
- daily
- several times daily

**5) Cognitive impairments**

16. I’ve often been misplacing things lately

- No
- Yes, sometimes
- yes, often, always
