# Supplementary material for: Caring for the elderly: A person-centered segmentation approach for exploring the association between health care needs, mental health care use, and costs in Germany
Source: PLoS One. 2019 Dec 19;14(12):e0226510. doi: 10.1371/journal.pone.0226510 (PMC6922348; doi:10.1371/journal.pone.0226510)
Supplement: S2 File — (DOCX) [file pone.0226510.s002.docx]

S2 Table: Comparison of model fit statistics

|  | BIC ( L^2^) | CAIC ( L^2^) | AIC ( L^2^) | AIC3 ( L^2^) | L^2^ | npar | df | p-value (L^2^) |
| --- | --- | --- | --- | --- | --- | --- | --- | --- |
| 1 Cluster model | -19925,1661 | -22889,1661 | -2105,5440 | -5069,5440 | 3822,4560 | 53 | 2964 | 7,9e-25 |
| 2 Cluster model | -20464,7022 | -23422,7022 | -2681,1522 | -5639,1522 | 3234,8478 | 59 | 2958 | 0,00023 |
| 3 Cluster model | -20521,3388 | -23473,3388 | -2773,8610 | -5725,8610 | 3130,1390 | 65 | 2952 | 0,011 |
| 4 Cluster model | -20539,5506 | -23485,5506 | -2828,1449 | -5774,1449 | 3063,8551 | 71 | 2946 | 0,064 |
| 5 Cluster model | -20509,8953 | -23449,8953 | -2834,5616 | -5774,5616 | 3045,4963 | 77 | 2940 | 0,086 |
| 6 Cluster model | -20489,1265 | -23423,1265 | -2849,8649 | -5783,8649 | 3018,1351 | 83 | 2934 | 0,14 |

Note: The model L^2^ statistic indicates the amount of the association among the variables that remains unexplained after estimating the model. The lower the value, the better the model fit to the data. In general, among the models with a p-value >0.05 the one that is most parsimonious would be selected (Vermont J & Magidson K, LatentGold 4.0 user’s guide). However, the five-cluster-solution shows a lower L^2^ compared to the 4-cluster-solution as well as a lower AIC and AIC3. In addition, a comparison test showed that the 5-cluster-solution provided a significant improvement over the four-cluster-solution. The interpretability of the clusters also indicated that the five-cluster-solution would be the optimal one.
